# The complete mitochondrial genome of *Tachysurus nitidus* (Siluriformes: Bagridae) from the Geum river in Korea

**DOI:** 10.1080/23802359.2021.1906181

**Published:** 2021-04-06

**Authors:** Yeong-Ho Kwak, Keun-Yong Kim, Ha-Yoon Song, Mi-Young Song

**Affiliations:** a Inland Fisheries Research Institute, National Institute of Fisheries Science, Gapyeong, Republic of Korea; b AquaGenTech Co., Ltd, Busan, Republic of Korea

**Keywords:** Bagridae, mitogenome, phylogenetic analysis, South Korea, *Tachysurus nitidus*

## Abstract

The complete mitochondrial genome of a bagrid catfish, *Tachysurus nitidus* was completely analyzed by the primer walking method. It was composed of 13 protein-coding genes, 2 ribosomal RNA genes, 22 transfer RNA genes, and a control region with a total length of 16,537 bp. In the phylogenetic tree, using mitochondrial genome of 13 related sequences revealed that *T. nitidus* (MW451217) of Korea is clustered with *T. nitidus* (KC822643) of China. This complete mitochondrial genome provides an important resource for reviewing the phylogenetic relationships and taxonomic status of the bagrid species.

The bagrid catfish (Siluriformes: Bagridae) is widely distributed in Africa and Asia, with about 221 species in 19 genera (Nelson et al. 2016). *Tachysurus nitidus* is a freshwater species belonging to the Bagridae family in Siluriformes order, which inhabits reservoirs, lakes, and large rivers. This species has commercially important values in East Asia (Kindong et al. 2019). In this study, we reported the complete mitochondrial DNA (mitogenome) sequence of *T*. *nitidus*, and examined the phylogenetic relationship in comparison with closely related species.

A specimen of *T*. *nitidus* was collected from the Geum River from Korea (36°9′57.81″N, 127°0′24.58″E) in October 2019. This specimen was currently deposited in the storage of the National Institute of Fisheries Science (Ha-Yoon Song, fish8607@korea.kr) under a voucher number, NFRDI-FI-TS-0054116. Genomic DNA was extracted using a piece of the pelvic fin tissue according to Asahida et al. (1996). The extracted genomic DNA was amplified through two long-range PCR (LC-PCR) amplification reactions, whose PCR products were sequenced according to the primer walking methods using 25 sequencing primers. The complete mitogenome sequence of *T. nitidus* was deposited in NCBI (GenBank Accession No. MW451217).

The mitogenome of *T. nitidus* was composed of 13 protein-coding genes (PCGs), 2 ribosomal RNA (rRNA) genes, 22 transfer RNA (tRNA) genes, and a control region (D-loop) with a total length of 16,537 bp. Its gene composition and organization were similar to those of typical vertebrates. Most PCGs began with a start codon ATG except the *CO1* gene, which initiated with GTG. There are nine PCGs terminated with a complete stop codon TAA, whereas the rest four, including the *CO2*, *CO3*, *ND4*, *COB* genes, ended with an incomplete one.

The mitogenome sequence of *T. nitidus* and allied species was analyzed with the Kimura 2-parameter model of the maximum likelihood (ML) method using MEGA version 7 software, and phylogenetic tree was constructed through bootstrap 1000 replicates (Kumar et al. 2016). The 12 mitogenome sequences were downloaded from GenBank in NCBI, and *Lebiasina astrigata* (NC015750) was used as an out group for the phylogenetic analysis (Figure 1).

**Figure 1.**
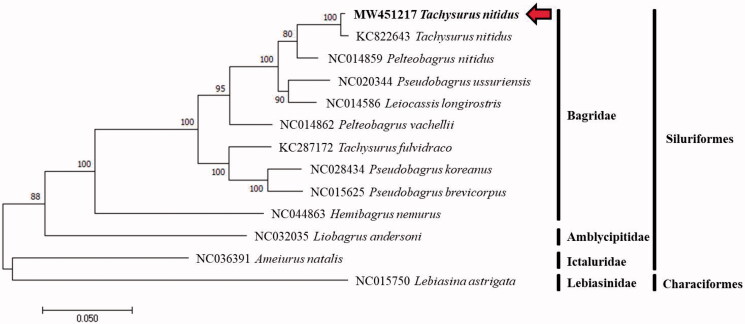
A phylogenetic tree reconstructed based on complete mitochondrial genome sequences from the orders Siluriformes and Characiformes. Analysis was using MEGA version 7 software with maximum likelihood method and bootstrap values 1000 replicates. Species analyzed in this study (MW451217) were in bold type and red arrow.

The phylogenetic tree showed that the *T. nitidus* from the Geum River formed a monophyletic group with the other bagrid species and clustered with the *T. nitidus* specimen (KC822643) from China. The phylogenetic results in this study revealed the *P. nitidus* specimen from China splits off the cluster of *T. niditus.*

Traditional fish classification based on the comparative morphological method remains controversial since in some cases, morphological features with small intraspecific differences provide limited value for the validity of a species (Boidya et al. 2015). For instance, the species *Leiocassis crassilabris* has been undergone many revisions and reclassifications until recently reidentified as a species of the genus *Tachysurus*, or the three genera *Pseudobagrus*, *Pelteobagrus*, and *Leiocassis* of the Bagridae were reported inconsistent and chaotic in taxonomic status (Lee and Kim 1990; Ng and Kottelat 2007; Zou et al. 2020). Therefore, the molecular method using a mitochondrial DNA fragment or a whole mitogenome has become an effective tool in fish identification and phylogenetic analysis (Li et al. 2020). In this study, the complete mitogenome of *T. nitidus* is disclosed which could provide baseline data for species identification and molecular phylogenetics, and effective management of the fisheries resources.

## Data Availability

The data that support the findings of this study are openly available in NCBI website (http://www.ncbi.nlm.nih.gov), reference number (MW451217).
